# Effects of 5-Year Interventions on Cardiovascular Risk Factors of Factories and Offies Employees of Isfahan and Najafabad: Worksite Intervention Project-Isfahan Healthy Heart Program

**Published:** 2010

**Authors:** Maryam Boshtam, Nizal Sarafzadegan, Karim Zare, Shahriar Sadeghi, Firoozeh Sajjadi, Katayoun Rabiei, Mansoreh Boshtam

**Affiliations:** 1 MSc, Animal Physiology, Isfahan Cardiovascular Research Center, Isfahan University of Medical Sciences, Isfahan, Iran; 2 MD; Professor of Cardiology,. Isfahan Cardiovascular Research Center, Isfahan University of Medical Sciences, Isfahan, Iran; 3 BS, Deputy of Health, Isfahan University of Medical Sciences, Isfahan, Iran; 4 BS, Isfahan Cardiovascular Research Center, Isfahan University of Medical Sciences, Isfahan, Iran; 5 MD, Isfahan Cardiovascular Research Center, Isfahan University of Medical Sciences, Isfahan, Iran

**Keywords:** Risk Factors, Cardiovascular Disease, Workplace, Intervention

## Abstract

**BACKGROUND:**

Effects of 5-year interventions of Worksite Intervention Project from Isfahan Healthy Heart Program on cardiovascular risk factors of factories and offices employees were studied in Isfahan and Najafabad (intervention area) compared to Arak (control area).

**METHODS:**

We had especial interventions for nutrition, physical activity and smoking as well as hypertension and obesity screening systems in all offices and factories, and other risk factors screening systems whenever possible. Before and after the interventions, questionnaires containing demographic and other required data were completed for the two populations; height, weight and blood pressure (BP) were measured and a fasting and 2h blood sample was taken for the measurement of blood sugar (BS) and lipid levels.

**RESULTS:**

The prevalence of hypercholesterolemia, hypertriglyceridemia and central obesity decreased, but low HDL increased in office staff (P < 0.01). Waist circumference, HDL and total cholesterol mean values decreased, and diastolic BP and fasting and 2h BS increased among the intervention group. In factory workers, the prevalence of hypertriglyceridemia and central obesity decreased, while low HDL prevalence increased in intervention group (P < 0.001). Mean values of waist circumference, HDL and total cholesterol, and triglyceride decreased significantly (P < 0.001), while diastolic BP and fasting BS increased.

**CONCLUSION:**

It seems that Worksite Intervention Project has a protective effect on CVD risk factors in factories and offices employees. So, the modifiable project can be used as an applicable tool for health improvement in worksites which creates tangible changes in employees’ lifestyle.

## Introduction

Cardiovascular diseases (CVDs) have an undeniable role in morbidity and mortality in developing countries.^1^ In our country too, half of mortality happens due to CVDs.^2^ Therefore, preventive strategies can considerably reduce CVDs’ mortality rate and economical loss.

It seems that worksites are appropriate places in which a huge population can be educated about health-related behaviors^3^, therefore, in some countries, competitive and consecutive models have been designed and used in health promotion activities.^4^

Evidence suggests that personal recommendations about CVDs to a vast number of workers can significantly reduce cardiovascular and stroke risk factors.^5^ This success may be attributed to some major reasons: first, adults, who make up a big section of the society, are included in the worker group and so they are easily accessible in worksites; second, interventions can be repeated in worksites, which makes it possible to make desirable changes in people with different levels of readiness. In addition since worksite-based interventions not only affect workers, but also the whole society, making overall corrections would be more likely to happen.^6^ Finally, there are many people in worksites who do not receive interventions in any other ways.

Considering the high prevalence of CVD risk factors in our country (7, 8), and the prevent-ability of them in worksites, Worksite Intervention Project (WIP), a part of Isfahan Healthy Heart Program (IHHP), has used factory and office workers in Isfahan and Najafabad as intervention group. The obtained results were compared to a control group (Arak employees) in order to get to a practical strategy for CVD prevention in Iranian worksites, a strategy which could also be used in societies culturally similar to Iran.

## Materials and Methods

WIP is an intervention project included in IHHP. It has been the first worksite intervention project in Iran and aimed to prevent CVDs and other non-communicable diseases and to correct lifestyle. WIP's interventions are easy and applicable, and don't need any extra budget. A number of elements have been considered while designing these interventions, some of which include “what behaviors need to be changed?”, “what is the purpose of changes?”, “how can the available facilities be used?”, “what changes are possible to be made in worksites?”, “how can the outcomes be used to encourage other worksites?” and finally, “how can we be sure about the outcomes?”.

An important quality of this project is that its executives didn't directly participate in worksites; rather, in each factory or organization, the directors recommended one of the personnel as the health liaison that had to lead and conduct all the interventions. The project's executives’ role was solely to design the interventions, prepare educational materials, educate health liaisons and supervise the conduction of the interventions.

Target groups in worksites were divided into two parts. In the offices, target groups were all the employees and managers and in the factories, office employees, workers and directors were included.

WIP's interventions were discussed thoroughly in an earlier article; 9 anyhow they briefly consisted of education on proper food, modification of food menus and ingredients along with cooking methods in factory and office restaurants, education on appropriate physical activity, planning on embedding physical activity and exercise in work hours, education on how harmful smoking is, and finally, prohibition of smoking as a rule. The screening systems for hypertension and obesity were designed to be used in all factories and offices. In worksites that were more interested, other risk factors were also monitored. However, in the final years of interventions, a law was passed which made periodical examination of workers, in factories with more than 50 personnel, obligatory, and therefore the screenings were performed through these examinations.

Educational material included a booklet, a number of pamphlets and posters, and IHHP's newsletters and educational CDs. The booklet was written in plain language and based on educational principles and contained up-to-date information.

The information about CVD risk factors, before and after the interventions, were obtained from the first and third phases of IHHP, in which a set of questionnaires containing demographic data, and also questions about people's disease history; knowledge, attitude and practice of nutrition, physical activity and smoking; hypertension, dyslipidemia, diabetes and obesity history were completed by educated interviewers for two independent samples (6400 people in the first phase and 4600 in the third phase).

In both phases, height, waist and hip circumfe rences were measured using a meter; weight was measured in light clothes and without shoes using a Seca scale; blood pressure was measured in sitting position from right arm twice, after at least 5 minutes of rest using a mercury manometer, and the mean value was recorded as the subject's blood pressure; a fasting blood sample (10 cc) was obtained from all subjects in order to measure fasting blood sugar (FBS), HDL, LDL and total cholesterol (HDL-C, LDL-C and TCho), and also triglyceride (TG) levels; to test the second-hour blood sugar level (2hpp), another blood sample was also taken 2 hours after eating glucose.

Serum lipids and FBS were measured by an ELAN autoanalyzer (Eppendorf, Germany), in the first phase, and by a Hitachi autoanalyzer (Model 902, Japan), in the third one. In both phases, Pars Azmoon kits (Iran) were used to measure serum lipids and Biosystems for blood sugars. IHHP's execution facts were completely explained elsewhere.^10^, ^11^

People with systolic blood pressure (SBP) >= 140 mmHg and/or diastolic blood pressure (DBP) >= 90 mmHg and/or using hypertension medications were considered hypertensive; subjects with FBS >= 126 mg/dl and/or 2hpp >= 200 mg/dl and/or using anti-diabetic medications were considered diabetic; those with serum TG >= 200 mg/dl were considered hypertriglyceridemic, and people with total cholesterol (TCho)>= 240 mg/dl were considered hypercholesterolemic. Low HDL-C was defined as HDL-C < 50 mg/dl in men and HDL-C < 40 mg/dl in women. High LDL was defined as LDL-C >= 160 mg/dl. People with BMI >= 25 Kg/m2 were known as obese or overweight. Waist circumference > 102 Cm for men and > 88Cm for women was defined as abdominal obesity.

This program was supposed to end in 2006, but due to financial problems, the third phase was postponed until 2007. Moreover, in 2005, the assessments in control group (Arak) were not conducted as a result of a lack in budget.

### Statistical Analysis

Obtained data entered and managed in an EPI file and analyzed using SPSS (version 15.5) software. Two Way ANOVA, Logistic regression and Chi Square tests used to compare CVD risk factors between two populations during 5 years of intervention.

## Results

The results of the study are presented in Tables 1–5.

**Table 1:** Factories and offices employees distribution in Isfahan and Najafabad (intervention area) and Arak (control area), based on sex and level of education, in IHHP, during 2001–2007

	Year	Number	Sex		Education (years)		

			Women No (%)	Men No (%)	0 – 5	6 – 12	>12
**Factory Workers**							
Intervention	2001	992	7(0.7)	985(99.3)	468(47.3)	493(49.8)	29(2.9)
	2007	725	11(1.5)	714(98.5)	188(26.0)	484(66.9)	51(7.1)
Control	2001	988	6(0.6)	982(99.4)	543(55.0)	428(43.4)	16(1.6)
	2007	947	9(1.0)	938(99.0)	419(44.2)	502(53.0)	26(2.7)
**Office Staff**							
Intervention	2001	616	120(19.5)	496(80.5)	57(9.3)	265(43.4)	288(47.2)
	2007	441	84(19.0)	357(81.0)	21(4.8)	126(28.6)	294(66.7)
Control	2001	590	84(14.2)	506(85.5)	54(9.2)	264(45.1)	267(45.6)
	2007	179	20(11.2)	159(88.8)	15(8.4)	84(46.9)	80(44.7)

**Table 2:** The prevalence* of CVD risk factors among factories workers of intervention and control groups, before and after intervention

Risk Factors	Intervention		Control		P

	2001	2007	2001	2007	

	No (%)	No (%)	No (%)	No (%)	
Hypertension	134(13.5)	88(12.5)	82(8.3)	98(10.3)	0.12
Diabetes	36(3.6)	39(4.5)	32(3.3)	37(3.9)	0.49
Hypercholesterolemia	154(7.15)	60(8.4)	124(12.9)	70(7.5)	0.66
Hypertriglyceridemia	325(33.1)	1333(18.6)	262(27.3)	228(28.3)	< 0.001
Low HDL	314(32.4)	356(49.8)	341(35.7)	397(42.5)	< 0.001
High LDL	114(12.6)	56(7.8)	94(10.6)	62(6.6)	0.95
Abdominal obesity	135(13.7)	69(9.9)	75(7.6)	71(10.9)	< 0.001
Overweight or Obesity	454(46.5)	342(49.2)	388(39.4)	381(40.3)	0.61

* The prevalence has been corrected for age and sex.

**Table 3:** The prevalence* of CVD risk factors among offices staff of intervention and control groups, before and after intervention

Risk Factors	Intervention		Control		P

	2001	2007	2001	2007	

	No (%)	No (%)	No (%)	No (%)	
Hypertension	73(11.9)	59(14.0)	63(10.7)	22(12.3)	0.93
Diabetes	20(3.3)	17(3.9)	16(2.7)	11(6.2)	0.2
Hypercholesterolemia	130(21.4)	37(8.6)	71(12.3)	22(12.4)	< 0.001
Hypertriglyceridemia	206(33.9)	89(20.6)	165(28.6)	46(26.0)	< 0.03
Low HDL	209(34.7)	231(53.6)	207(35.7)	67(37.9)	< 0.001
High LDL	88(15.4)	41(9.5)	55(10.4)	15(8.5)	0.37
Abdominal obesity	146(23.8)	70(16.9)	77(13.1)	24(17.0)	< 0.001
Overweight or Obesity	315(51.8)	219(52.2)	281(47.7)	98(55.1)	0.24

* The prevalence has been corrected for age and sex.

**Table 4:** Mean ±SD of CVD risk factors among the factories workers of the intervention and control groups, before and after intervention

	Intervention		Control		P

	2001	2007	2001	2007	

	Mean ±SD	Mean ±SD	Mean ±SD	Mean ±SD	
Waist circumference (cm)	90.0 ± 11.0	89.7 ± 11.5	86.1 ± 11.6	88.7 ± 11.5	< 0.001
Body Mass Index (Kg/m2)	24.8 ± 4.3	25.1 ± 4.2	24.3 ± 4.0	72.6 ± 10.2	0.54
Diastolic Blood Pressure(mmHg)	74.5 ± 10.6	75.2 ± 9.9	74.8 ± 8.9	112.0 ± 14.9	< 0.001
Systolic Blood Pressure(mmHg)	15.3 ± 15.8	113.1 ± 15.4	112.4 ± 14.9	111.6 ± 32.1	0.69
LDL Cholesterol (mg/dl)	113.7 ± 39.8	113.9 ± 32.3	109.7 ± 38.5	42.3 ± 10.3	0.51
HDL Cholesterol (mg/dl)	44.9 ± 9.6	40.9 ± 9.1	44.4 ± 9.9	42.3 ± 10.3	0.003
Triglyceride (mg/dl)	187.2 ± 122.6	147.5 ± 101.0	177.1 ± 126.0	163.8 ± 98.8	0.001
Total Cholesterol (mg/dl)	194.6 ± 48.3	184.0 ± 39.0	187.0 ± 45.1	186.8 ± 38.9	< 0.001
2hpp (mg/dl)	91.7 ± 40.9	97.8 ± 27.6	93.6 ± 36.2	97.9 ± 30.5	0.45
FBS (mg/dl)	81.7 ± 28.3	91.0 ± 24.5	82.4 ± 21.6	84.6 ± 17.2	< 0.001

**Table 5:** Mean ±SD of CVD risk factors among the offices staff of the intervention and control groups before and after intervention

	Intervention		Control		P

	2001	2007	2001	2007	

	Mean ±SD	Mean ±SD	Mean ±SD	Mean ±SD		
Waist circumference (cm)	90.8 ± 0.46	88.8 ± 0.55	88.4 ± 0.49	90.4 ± 11.3	0.003
Body Mass Index (Kg/m2)	25.5 ± 0.17	25.9 ± 0.2	25.5 ± 0.18	25.2 ± 3.8	0.67
Diastolic Blood Pressure(mmHg)	73.2 ± 0.43	74.4 ± 0.51	75.5 ± 0.46	73.3 ± 9.9	< 0.001
Systolic Blood Pressure(mmHg)	111.7 ± 0.6	110.2 ± 0.76	112.0 ± 0.68	112.4 ± 14.4	0.82
LDL Cholesterol (mg/dl)	119.8 ± 1.6	116.6 ± 1.8	110.3 ± 1.7	115.5 ± 38.9	0.03
HDL Cholesterol (mg/dl)	47.4 ± 0.44	42.8 ± 0.52	47.1 ± 0.47	44.0 ± 9.9	0.003
Triglyceride (mg/dl)	170.7 ± 4.9	139.2 ± 5.7	160.8 ± 5.2	162.6 ± 10.6	0.16
Total Cholesterol (mg/dl)	200.5 ± 1.8	187.4 ± 2.1	187.6 ± 1.9	191.0 ± 38.6	< 0.001
2hpp (mg/dl)	93.9 ± 0.81	99.1 ± 1.8	93.6 ± 1.6	109.0 ± 37.8	0.002
FBS (mg/dl)	78.9 ± 0.81	91.0 ± 24.5	80.4 ± 0.86	86.5 ± 21.3	0.047

As Table 1 shows, in both regions, sex distribution among the workers was the same during the two phases, but the percentage of women in intervention group was higher than control group. However, in both populations, the percentage of women among factory workers was very low, but higher among office workers.

As Table 2 suggests about the factory workers, while the prevalence of low HDL-C in intervention group had a significant increase compared with the control group (P = 0.002), the prevalence of hypertension was significantly reduced in the first group (P=0.04), and the same results were also seen about the prevalence of abdominal obesity and hypertriglyceridemia (P < 0.001). Interventions didn't make any significant changes in other risk factors among workers (P > 0.05).

In case of office workers, a significant reduction in the prevalence of hypercholesterolemia (P < 0.001), hypertriglyceridemia (P = 0.024), and abdominal obesity (P = 0.006) was observed in intervention group as compared with the control group (Table 3). On the contrary, the prevalence of low HDL-C significantly increased in intervention group. No significant change, between the two regions, was found in other risk factors (P > 0.05).

Table 4 shows the changes in the mean value of CVD risk factors as a result of interventions. The mean values for waist circumference (P < 0.001), HDL-C (P=0.003), TG (P=0.001) and total cholesterol (P < 0.001) were significantly reduced in the workers of intervention group as compared with the control group. However, the mean values for DBP (P < 0.001) and FBS (P < 0.001) had a significant increase among the intervention group.

As a result of interventions, similar changes regarding waist circumference (p = 0.003), DBP (P < 0.001), HDL-C (p = 0.003), TCho (P < 0.001) and FBS (p = 0.047) were obtained among office workers in the two groups (Table 5), but the mean value for LDL-C (p = 0.03) in intervention group was significantly less than that of the control group, and the increment in the mean value for 2hpp was significantly more in the control group (P = 0.002).

## Discussion

In general, WIP's 5-year interventional activities in workplaces have been effective in reduction of CVD risk factors; therefore it can be suggested that the prevalence of CVDs among office and factory workers will reduce in the future.

A major CVD risk factor is hypertriglyceridemia, the prevalence of which was significantly reduced in workers of intervention group, while the opposite happened in the control group. This outcome confirms that the nutritional interventions and the efforts to change the food habits were satisfactorily effective. A research on nutritional changes in intervention group revealed that hydrogenated vegetable oils were used significantly less among this group (as compared with control group), while the consumption of fruit and vegetables was increased.^12^

Similarly, abdominal obesity reduced in workers of intervention group, but rose among the control. group. It is known inappropriate food and insufficient physical activity are two reasons that lead to obesity and as it seems proper changes in workers’ food habits^12^, along with stretching exercises at workplace and altering the workers’ routine activities into beneficial ones led to the mentioned improvement in intervention group.

However, it is surprising that the prevalence of low HDL-C was increased among intervention group after the interventions since we know that the most effective factor on HDL-C level is physical activity and an increment in this factor is associated with an increase in HDL-C serum level.^13^ So, the increased prevalence of low HDL-C and the decreased serum level of HDL-C would probably be related to the significant reduction in Tchol in intervention group because when Tchol is decreased as a result of dietary changes, without any noticeable increment in physical activity, HDL-C level is reduced. Similar results were also obtained in a study conducted by Geil et al. in which only nutritional interventions were implemented.^14^ But in a study performed by Lan Londe et al., where the nutritional interventions were accompanied with physical activity related interventions, Tchol to HDL-C ratio improved.^14^ It's worth mentioning that the reduction of TG and Tchol, as a result of interventions, among factory workers indicate the positive effects of nutritional interventions.

Although a small increase in the prevalence of central obesity among both groups existed, the difference was not significant. This shows that population's weight didn't have a considerable change, but their body size and shape were improved; a result which observed among the whole society, too .^15^ It seems that abdominal obesity changes might be due to dietary changes, and since they were not accompanied with physical activity increment, weight reduction was not observed. On the other hand, the prevalence of smoking also decreased among factory and office workers, which can, in turn, be responsible for BMI increase among them (because we know that the appetite grows after a person quits smoking or smokes less).

As the results of this study suggest, the prevalence of hypertension, in intervention group, didn't change significantly when compared with the control group; the mean value for DBP significantly increased among intervention group, and decreased among the control group. However, SBP changes were in favor of interventions.

One nutritional intervention used in WIP was to educate people to use less salt; restaurant chefs were also told to do the same when cooking.^9^ Given that hypertension is directly related with salt consumption, the outcomes obviously reflected the effectiveness of interventions. On the other hand, only SBP is directly related with salt consumption, and DBP is associated with peripheral resistance, so, the obtained results seem reasonable. The given data is also in agreement with IHHP results for the whole society.^16^

Blood sugar increased in two studied groups, but significantly more in intervention group. This may be attributable to the replacement of fats with some starches, or more importantly to age increment especially among intervention group.^16^ However, changes in FBS levels are not normally considered a major index of intervention effectiveness.

Most office workers have a little physical activity during office hours and spend many hours sitting. Besides, except for a few organizations, most organizations close at 2 p.m., and hence no food is served in them and their staff eat food at home or somewhere out of the workplace. Therefore, most interventions were just educational. As the results of the present study show, among the office workers of intervention group, the prevalence of hypertriglyceridemia and hypercholesterolemia significantly decreased, while the prevalence of low HDL-C was considerably increased. Furthermore, similar to factory workers, interventions led to a significant reduction in the prevalence of abdominal obesity, but didn't cause any noticeable changes in central obesity. Since there was a great dietary change among the office workers, in the intervention group, without any particular effort from the project, it seems that the education level had a substantial effect on the reduction of blood lipids in these people. Moreover, it's usually women who make food, so the more percentage of women working in offices than those working in factories could be another reason for the effectiveness and practicality of the educational programs. Changes in risk factors were similar to those observed in the whole society, which shows that this age group follows the social changes.^15^

The prevalence of hypertension increased significantly in both intervention and control groups, which is in contrast with the results of our previous report.^15^ The only significant difference was in case of DBP for which an increase in the intervention group and a decrease in the control group were observed. So it can be inferred that these people didn't change the amount of salt consumption; however, other factors such as regular exercise, smoking, and family history can also affect hypertension.

Considering observed changes among office workers, it could be concluded that the interventions were most effective on food habits, whereas they hadn't an appropriate effect on physical activity.

A clinical study in the U.S. showed that after 2 years of interventions, especially physical activity related educational interventions, weight, blood lipids levels and hypertension were not changed significantly.^17^

In a similar study in Malaysia, 2 years of workplace interventions made a significant reduction in total cholesterol among the intervention group; HDL-C was reduced in the intervention and the control groups, but this reduction was significantly more in the intervention group; no significant change in TG, FBS and BMI was observed.^14^

Another experience in Japan indicated that 18 months of comprehensive interventions on office worksites significantly reduced BMI, SBP, Tchol and TG among the intervention group. In addition, when subjects with one special risk factor were considered separately, a significant reduction of BMI, TG and Tchol was also seen in them.^18^

The results of HIPOP-OHP study, which conducted 4 years of physical activity related interventions in labor worksites, showed a significant increase in HDL-C levels in the intervention group.^19^ However, worksite chronic disease prevention program suggested that even 6-week or 6-month interventions can increase health knowledge, improve nutrition and physical activity, and also decrease many health risks among the employees .^21^

Another study indicated that low-intensity; short-term interventions in worksites can significantly improve health behaviors, nutritional knowledge, and decrease SBP in the intervention group. Nevertheless, this study suggested that a longer duration or more intensive intervention may be required to achieve further reduction in risk factors.^21^

In another experience, just Tchol and HDL-C were reduced significantly after interventions.^22^

In the interventions conducted in IMPACT study in the USA, a significant reduction in Tchol was seen among hypertriglyceridemic subjects.^23^

A review article, which reviewed various studies on white European and American factory and office workers, reported a 5-9% reduction in Tchol as a result of worksite nutritional interventions.^24^

Generally, the present study suggested that worksite interventions would be effective on CVD risk factors. As the scientists believe, management support and the feeling of ownership among the participants are essential for the project effectiveness^4^, similarly in this study, the participation of worksite executives and workers contributed to the project's success.

Furthermore, the simultaneous of this project with a number of other interventional projects on different target groups makes those other interventions more effective.

In addition to group educational programs, this project featured some personal counseling for high risk subjects. Research shows that these kinds of interventions have been effective in worksite health promotion .^25^

Therefore, it can be concluded that WIP, as a practical tool for health promotion among factory and office workers in their worksites, creates tangible changes in people's lifestyle which not only ensures healthy workers, but also leads to a healthy societycells.^9^, ^11^–^13^ The results of this study which are indicative of relative reducing in negative surface charge of LDL particle in the presence of CRP, are in line with the results of a study conducted by Rufail et al. It is noteworthy that in that study, the result of electrophoresis was obtained only after 2 hours of incubation, but in the present study, this period was extended to 18 hours.^19^ It can be explained that possibly this protein participates in atherogenesis through a mechanism leading to conformational changes in the semi-oxidized LDL molecule.

The results of this study are consistent with recent studies which have highlighted a kind of relationship and physical binding between CRP and Ox-LDL.^7^, ^8^, ^20^ It is possible that binding of CRP to some areas of the LDL particle (like, phosphocholine moiety) leads to some changes on the surface of this lipoprotein, resulting in reducing of susceptibility of LDL to more oxidation in addition to preparing this modified-lipoprotein for phagocytosis by macrophage receptors.

In the present study, different level of CRP within its physiological range in serum were used to assess the degree to which LDL oxidation would be influenced in progressive concentrations (0 µg/ml, 0.5 µg/ml and 2 µg/ml CRP) because according to previous studies, it has been clearly demonstrated that the elevated level of CRP is related to higher incidence of cardiovascular disease (17, 18, 22). It is notable that the results of some recent studies have shown that vascular endothelial cells are prompted by a number of trigger factors to secrete CRP^22^ this finding can form a basis for the hypothesis that this protein may be dispatched to the external space, where LDL is invaded by oxidative factors. Also one could hypothesize that LDL, which in the initial stages of oxidation induces CRP secretion, indirectly recalling this protein towards itself to resist invading factors. Based on the result of this study, maybe the processes hypothetically ascribed to CRP be inherently conducive to vascular health and this protein may have antiatherogenic properties by providing relative protection for LDL against oxidation, but it must be borne in mind that it may be possible that, under acute conditions,it act to expedite atherogenesis (such as; cooperation with macrophages to form foam cells). This second hypothesis may be more compatible with the fact that the level of CRP increases during cardiovascular diseases.^9^, ^11^, ^12^, ^19^
